# Durability and damage resistance of sustainable Portland limestone cement-based (ultra) high-performance concrete in seawater

**DOI:** 10.1617/s11527-025-02824-5

**Published:** 2025-10-26

**Authors:** Luca Galli, Prannoy Suraneni

**Affiliations:** Civil and Architectural Engineering, University of Miami, Coral Gables, FL 33146 USA

**Keywords:** Durability, Resilience, Seawater exposure, Supplementary cementitious materials, Ultra high-performance concrete

## Abstract

Coastal communities often rely on conventional reinforced concrete infrastructure to offer protection from the marine environment. However, these structures are highly susceptible to chloride-induced corrosion, compromising their durability. Ultra-high-performance concrete (UHPC) offers superior durability, but faces adoption barriers due to rigid design requirements and high costs, largely driven by steel fiber reinforcement. Recent advances in fiber technology and the development of greener cementitious materials created opportunities for alternative, sustainable, and cost-effective UHPC designs that could significantly enhance coastal infrastructure resilience, particularly as climate change intensifies natural hazards. However, data on the mechanical and long-term performance of such UHPC in harsh environments remain limited. This study investigates the durability and damage resistance of sustainable UHPC mixtures based on Portland limestone cement (PLC), incorporating high volumes of fly ash and slag, and reinforced with steel and polyethylene fibers. Specimens were exposed to seawater for up to 550 days and compared to a commercial UHPC. Evaluations included mass variation, electrical resistivity, ultrasonic pulse velocity, compressive strength, water sorption, thermogravimetric analysis, and resistance to chloride ingress and carbonation. Results indicate that the laboratory-developed UHPC mixtures, whether damaged or undamaged, exhibited excellent resistance to marine exposure, showing limited degradation, and performing comparably to the commercial UHPC. No significant chloride ingress or carbonation was observed in any mixture, regardless of fiber type.

**Supplementary Information:**

The online version contains supplementary material available at 10.1617/s11527-025-02824-5.

## Introduction

Reinforced concrete has been an indispensable material in marine construction during the last century, serving as the backbone for infrastructure such as ports, harbors, offshore platforms, breakwaters, and seawalls. Its adaptability, robustness, and cost-effectiveness made it the material of choice for structures designed to endure the harsh and unpredictable conditions of marine environments [1]. However, as these structures age, significant deterioration compromises their structural performance and reliability, threatening the safety and resilience of coastal communities. This is particularly concerning given that nearly 14% of the US shoreline (i.e., 23,000 km) is already covered with concrete infrastructure. Deterioration often manifests as cracking, spalling, and reinforcement corrosion. One of the most prevalent forms of degradation in such environments is chloride-induced corrosion of embedded steel reinforcement. Chloride ions from seawater penetrate the concrete cover, initiating corrosion processes that weaken the steel and generate expansive forces, leading to cracking and damage in the concrete matrix, in addition to loss of cover. Sulfate attack, alkali-aggregate reactions, and freeze–thaw are additional factors that exacerbate the degradation of concrete in coastal environments [2–4]. In recent decades, extensive research has been conducted on chloride transport and the service life of reinforced concrete structures [5–8]. However, the impacts of climate change have introduced additional complexities that are not totally understood. Rising global temperatures and changing climate patterns have amplified challenges faced by marine infrastructure. Extreme temperatures and precipitation are becoming more frequent and intense, while hurricanes, tornadoes, and winter storms are undergoing significant changes attributable to climate change [9, 10]. Zhang et al. [11] and Castro-Borges et al. [12] recently highlighted this problem. Others projected that, by the end of the twenty-first century, rising global surface temperatures and humidity could accelerate corrosion initiation rates by 2–18% in coastal areas and lead to premature structural failures by 3–31%, reducing a 100-year design life by an average of 15 years [13–15]. Additionally, carbonation lowers the pH of concrete, further decreasing the chloride threshold for steel corrosion, which intensifies the issue [16].

The American Society of Civil Engineers estimates that over $25 billion is required to improve and maintain the US marine infrastructure, including seawalls and levees, with a substantial funding gap persisting over the next decade [17]. Addressing these challenges requires a deeper understanding of climate change impacts to improve the design of both new and existing structures, ultimately reducing future repair costs. These demands necessitate the development of advanced materials with enhanced durability, lower maintenance needs, and optimized usage to meet the evolving requirements of resilient marine infrastructure.

Ultra-high performance concrete (UHPC) has the potential to become a possible solution. UHPC was introduced in the late twentieth century to produce concrete with enhanced durability and mechanical properties compared to conventional concrete. The improved properties of UHPC are a result of mixture proportions and ingredients, which ensure mechanical homogeneity and maximum particle packing density [18–21]. Although UHPC is widely recognized for its intrinsic durability, there are limited experimental studies in the literature that investigate its long-term performance under harsh conditions. Moffatt et al. [22] demonstrated the exceptional durability of UHPC exposed at tidal zone of marine environments for up to 21 years, where the UHPC prisms displayed only minimal surface damage, regardless of curing treatment or water-to-cement ratio. More recently, however, Zeng et al. [23] indicated slight performance reductions and superficial degradation due to steel fiber corrosion for UHPC specimens under artificial seawater exposure at 60 °C for 12 months, reinforcing the necessity for more studies on the topic. As the cement industry shifts toward greener alternatives such as Portland limestone cement (PLC), there is a growing need to develop newer UHPC formulations based on more sustainable binders. However, the sustainability of UHPC extends beyond its cementitious components. High volumes of steel fibers and chemical admixtures remain significant contributors to both the environmental footprint and overall cost of UHPC, making their optimization essential for achieving truly sustainable solutions. Advancing UHPC toward greater sustainability and cost-effectiveness, while relying on widely available materials, would not only reduce its environmental impact but also broaden its practical applications. Moreover, to meet the growing demand for resilient infrastructure in coastal regions, it is critical to evaluate the long-term performance of these new UHPC formulations to fully realize their potential.

This work aims to evaluate the durability of a sustainable PLC-based (ultra) high-performance concrete in seawater. In doing so, it also explores the partial or complete replacement of steel fibers with polyethylene fibers, which offer key advantages such as lower weight and resistance to chloride-induced corrosion. The novelty of this study lies in the comprehensive assessment of multiple properties of intact and damaged specimens submerged in seawater for one and a half years. By measuring mechanical strength, chloride penetration, microstructural changes, and other durability indicators, we provide valuable insights into the long-term performance of PLC based UHPC in marine environments. We not only address the durability of this material but also investigate its resilience. The findings have the potential to influence material selection and design practices in marine construction, promoting the use of more durable and environmentally friendly materials. Ultimately, the work aims to enhance the resilience of marine infrastructure, reduce maintenance costs, and mitigate the environmental impact associated with the construction and repair of marine concrete structures.

## Materials and methods

### Materials

The binder materials included a PLC complying with ASTM C595 type IL, with 14% limestone content, an ‘ultra-fine’ limestone (UL), an ASTM C618 Class F fly ash (FA), and an ASTM C989 ground granulated blast furnace slag (SL). The chemical compositions and the d_50_ of the cementitious materials used in the UHPC formulations were obtained from X-ray fluorescence (XRF) analysis and laser particle size diffraction testing and are reported in Table 1.

**Table 1:** Chemical composition (mass %) and d_50_ (μm) of the cementitious materials used [24]

Material	SiO_2_	Al_2_O_3_	Fe_2_O_3_	CaO	MgO	SO_3_	Na_2_O	d_50_
PLC	19.1	4.3	3.3	63.8	0.8	2.5	0.1	12.3
UL	5.5	2.9	1.3	49.6	0.5	0.1	0.1	1.4
FA	49.7	19.6	14.8	6.2	1.0	2.3	0.9	15.6
SL	33.8	14.5	0.7	41.6	5.7	3.0	–	13.5

The only aggregate used in the binder was mason sand with particle sizes ranging from 360–1600 μm. The UHPC formulations were fiber-reinforced through two types of reinforcing fiber materials: steel and polyethylene. Steel fibers (Sf), known for their ability to enhance UHPC’s mechanical properties, are common but increase costs significantly and are potentially subject to corrosion. To address this, polyethylene fibers (Pf) were explored as a partial or full replacement for steel. PE fibers are non-corrosive, making them ideal for chloride-rich environments. Moreover, research has shown that PE fibers benefit the strain-hardening behavior of UHPC, achieving tensile strain capacities up to 8%, which is substantially higher than the 0.1–1.0% typical of steel fiber-reinforced UHPC [25]. This increased ductility is critical for resisting crack propagation and maintaining durability under marine conditions. A total fiber content of 2% by volume was used for each mixture, using either 2% Sf, 2% Pf, or a hybrid combination of 1% Sf and 1% Pf. Fiber properties are described in Table 2.

**Table 2:** Main properties of the reinforcing fiber types

Type	Diameter (μm)	Length (mm)	Aspect ratio (L/ø)	Density (g/cm^3^)	Tensile strength (MPa)	Modulus of elasticity (GPa)
Steel fibers (Sf)	200	13	65	7.8	2750	200
Polyethylene fibers (Pf)	18	13	722	0.97	2700	114

Two high-efficiency, low-addition rate polycarboxylate-based high-range water reducers (HRWRs), compliant with ASTM C494, were used for the UHPC formulations. The commercial UHPC was provided with its dedicated HRWR, while the other was used for the lab-developed (U)HPCs. Table 3 reports the final mixture designs of the developed sustainable formulations and the commercial UHPC.

**Table 3:** Mixture designs of the tested formulation (kg/m^3^)

Mixture	Dry mix UHPC	PLC	UL	FA	SL	Sand	HRWR	Water	Sf	Pf
Comm. UHPC	2170	–	–	–	–	–	23	206	153	-
10UL-20FA-Sf	–	714	103	205	–	1022	20	205	157	-
10UL-20FA-SPf	–	714	103	205	–	1022	20	205	78	10
10UL-20FA-Pf	–	714	103	205	–	1022	20	205	–	20
10UL-25SL-Pf	–	664	103	–	255	1022	20	205	–	20
10UL-pf	–	919	103	–	–	1022	20	205	–	20

### Methods

#### Mixing procedures and exposure condition

Concrete mixing was carried out using a high-shear mixer with multiple blades to ensure thorough homogenization, minimize fiber segregation, and control air entrainment. Initially, 70% of the dry components (cement, sand, and supplementary cementitious materials) were blended at low speed for 2 min. Separately, 90% of the water and the high-range water reducer (HRWR) were pre-mixed for 2 min to ensure even HRWR distribution. This solution was then gradually introduced to the dry mixture over 1 min, with mixing continued at medium speed for full incorporation. Once the mixture became flowable, the remaining dry materials and the remaining water-HRWR solution were slowly added. The UHPC was mixed for an additional 7 min to achieve a consistent, flowable blend. Following a preliminary flow test to confirm satisfactory rheology, reinforcing fibers were added, and the mixture was blended for another 2 min to ensure uniform fiber distribution. Rheological properties were then assessed in accordance with ASTM C1856, and the flow test results are reported in Table S1 (Supplementary Material). Afterward, the UHPC samples were cast into plastic 50 mm cubic molds, consolidated by low-frequency vibration on a laboratory table for 30 s, and sealed to prevent moisture loss. After 24 h, curing proceeded in a monitored moist room compliant with ASTM C511 at 20 °C and ≥ 95% relative humidity until the end of the curing period. After 28 days of curing, the specimens were removed from the moist room. A portion was tested, including compressive strength assessment, as part of the experimental program. Another portion was damaged by loading the UHPC cubes to 75% of the average 28-day compressive strength recorded for each mixture. These specimens refer to the ‘damaged’ condition mentioned in this paper. The remaining undamaged specimens, together with the damaged ones, were then exposed to seawater at room temperature under stationary conditions. The exposure lasted 365 days for the damaged cubes and 550 days for the undamaged ones. The seawater chemical composition is reported in Table S2. The solution was periodically renewed every 3 months during exposure to maintain a pH of approximately 8.

#### Mass variation

The leaching behavior and degradation of 50 mm UHPC cubes were measured at 28, 365, and 550 days. These effects were in part evaluated by measuring the mass evolution during the curing period or the seawater exposure of the samples. For each mixture, three samples were extracted from the exposure chamber, prepared by moist wiping to achieve a quasi-saturated/saturated surface dry (SSD) condition, and subsequently tested.

#### Bulk resistivity and ultrasonic pulse velocity (UPV)

The bulk electrical resistivity and UPV measurements were conducted on the 50 mm UHPC cubes at 28, 365, and 550 days post seawater exposure. Bulk resistivity testing followed ASTM C1876, utilizing a bulk resistivity meter operating at 1 kHz. UPV testing was conducted through direct transmission with an ultrasonic device at a frequency of 54 Hz, in accordance with ASTM C597. For each mixture, three samples were removed from the exposure chamber, wiped with a moist cloth, and tested in a quasi-saturated/saturated surface dry (SSD) condition to ensure consistent, reliable measurements for comparative analysis. Necessary adjustments for sample size and dimensions were applied to accurately calculate electrical resistivity. Three replicate specimens were tested per mixture.

#### Compressive strength

The compressive strength test was investigated on the 50 mm UHPC cubes at 28, 365, and 550 days. The 50 mm UHPC cubes were taken from the moisture chamber and wiped with a moist cloth to be tested on a mechanical testing machine at a 2580 ± 133 N/s loading rate. A minimum of three samples were tested at each age. The test specimens were meticulously fabricated and prepared, ensuring the load application surfaces’ flatness. Plastic 50 mm cubic molds were used to accommodate the elevated number of specimens tested. Effects of using different types of mold materials and challenges in accurately and reliably verifying the compressive strength of high-strength concretes are known and reported in the literature [24, 26–28]. Considering these findings, secondary adjustments to the results presented in this paper may be admissible.

#### Silver nitrate and phenolphthalein tests

The evaluation of the ingress of deteriorating substances in the concrete specimens was measured by colorimetric methods. The silver nitrate technique consists of spraying 0.1 mol/l silver nitrate (AgNO_3_) solution over a recently fractured concrete sample. Silver nitrate spray solution is largely used to detect chloride depth penetration in concrete [29–31]. The phenolphthalein spray solution is a technique widely adopted to measure carbonation depth in concrete [32–34]. Chloride ingress and carbonation often occur simultaneously in many reinforced concrete structures. Numerous studies have investigated the combined effects of carbonation and chloride ions penetration in concrete [35–37]. For these reasons, shortly after the compressive strength test, the fractured UHPC cubes were split into two halves, and both solutions were sprayed on the surface of each sample to evaluate the ingress profiles of the aggressive substances after the specimens’ exposure to seawater.

#### Water sorption

The water absorption test was conducted to evaluate the relative susceptibility of undamaged and damaged UHPC specimens to moisture uptake. From each concrete cube, four samples (approximately 5 g each) were extracted from both the surface and the bulk after the compressive strength test. The initial mass of each sample was recorded before drying in a ventilated oven at 105 °C for two days. Following this initial drying period, and in accordance with ASTM C642, the mass was measured every 24 h until the difference between two consecutive measurements did not exceed 0.5% of the lesser value.

#### Thermogravimetric analysis

Thermogravimetric analysis (TGA) was conducted in accordance with ASTM C1872 to measure calcium hydroxide (Ca(OH)_2_) and calcium carbonate (CaCO_3_) content. The test was performed at ages 28, 365, and 550 days on approximately 50 mg of powder of samples taken from the surface and the bulk of the fractured concrete cubes of each mixture. The temperature was ramped at a rate of 10 °C/min from ambient temperature to 1000 °C in a nitrogen-purged atmosphere. Tests were generally done on one specimen for each mixture, and a tangential baseline method was used to calculate compound content values from the thermogravimetric curves.

## Results and discussion

### Mass variation

**Fig. 1:**
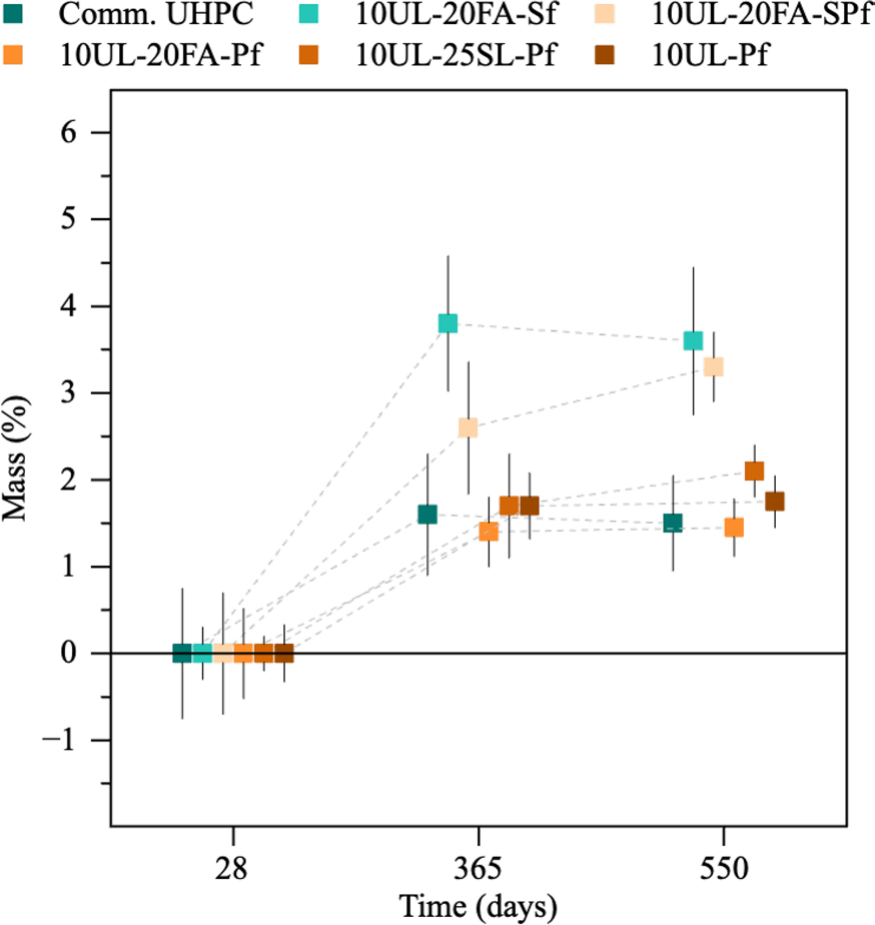
Mass evolution of undamaged UHPC cubes over time. The error bars shown in this figure and all other figures are two standard deviations of the mean

**Fig. 2:**
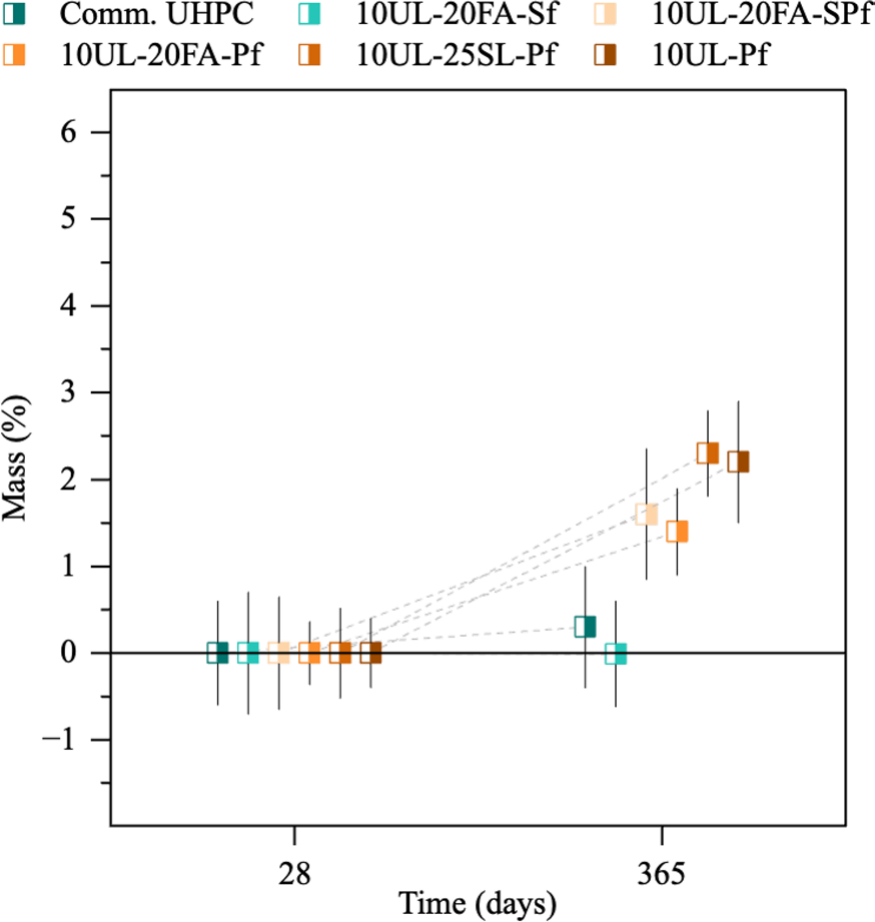
Mass evolution of damaged UHPC cubes over time

Figures 1 and 2 illustrate the mass variation of UHPC cubes under different conditions. Figure 1 presents the mass changes of undamaged UHPC specimens at 28 days, and after 365 and 550 days of seawater exposure. Values are shown as mean with error bars being two standard deviations of the mean. Figure 2 shows the mass variation of damaged UHPC cubes at 28 days and after 365 days of exposure. Overall, the specimens exhibited minimal mass alterations during seawater exposure, regardless of their undamaged or damaged condition. This mass change is likely the combined effect of multiple mechanisms, including the leaching of calcium hydroxide, C-S–H, and other hydrates, as well as water ingress, all acting concurrently to influence the observed values. During the early stages of seawater exposure, leaching was identified through localized areas of efflorescence on the surface of the different concrete cubes. However, no overall mass loss was observed at 365 days for any of the mixtures. Instead, the mass of undamaged UHPC mixtures with Sf increased by 1.6%, 3.8%, and 2.6% for the commercial UHPC, 10UL-20FA-Sf, and 10UL-20FA-SPf, respectively. Mixtures reinforced solely with Pf exhibited lower mass increases, with 10UL-25SL-Pf showing a maximum of 1.7%. The damaged specimens recorded mass increases of no more than 2.3% during the same period. Among these, the mixtures including Pf showed similar values to the undamaged specimens. The damaged cubes with only Sf recorded an increase of 0.3% for the commercial UHPC, while no mass increase was recorded for 10UL-20FA-Sf. The absence of mass increase of the damaged specimens, compared to the undamaged ones, is likely due to cracks formed during the damaging process, which caused small concrete fragments to detach from the steel fibers and be lost during seawater exposure, and due to greater leaching in the cracked specimen. All observed mass increases can generally be attributed to the gradual saturation of the specimens, even though the degree of saturation in UHPC likely remains limited due to its inherently low porosity. Notably, between 365 and 550 days, the rate of mass increase slowed across the undamaged mixtures, suggesting that the ingress and egress phenomena had reached a stabilization point. The presence of reinforcing fibers may have contributed to mitigating mass changes. Studies have shown that a 2% steel fiber content is highly effective in enhancing this effect [38] with Abbas et al. [39] reporting a reduction in pore volume as fiber content increases, thereby limiting water ingress and leaching from the cementitious matrix. This trend also applies to polyethylene fibers, where the combination of fiber content and its hydrophobic behavior likely contributed to further reducing mass gain in these specimens [40, 41].

### Bulk resistivity and ultrasonic pulse velocity

**Fig. 3:**
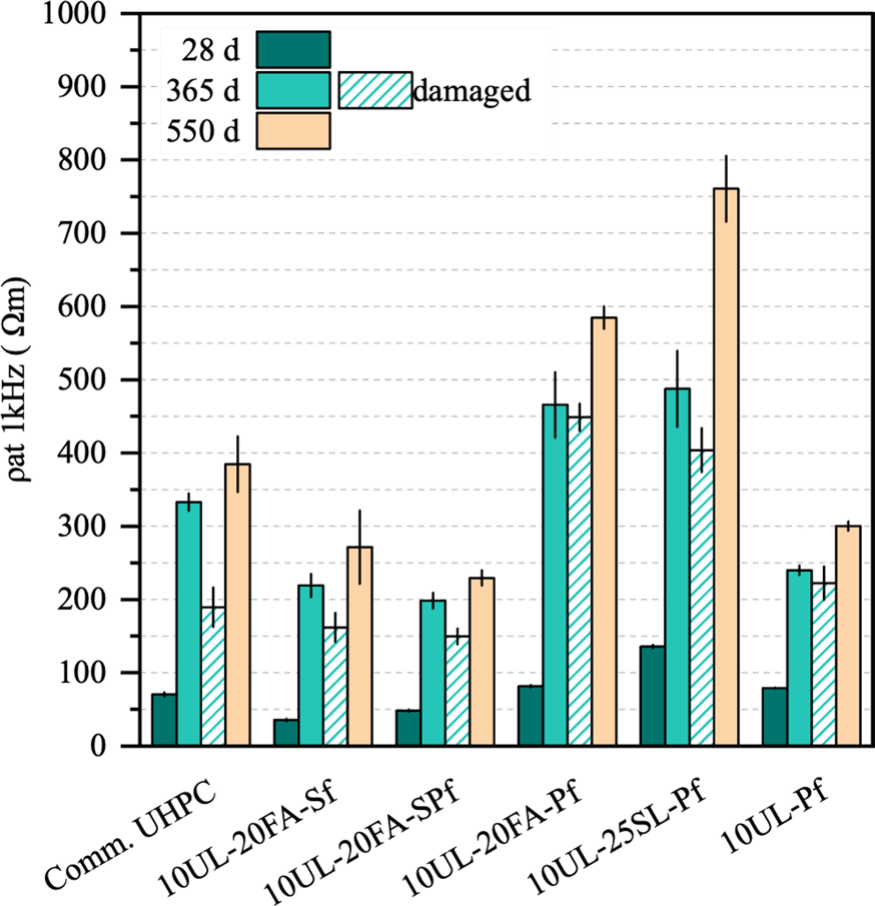
Electrical resistivity results of undamaged and damaged specimens at 28 days and after 365 and 550 days of exposure to seawater

Figure 3 presents the bulk resistivity results of the undamaged and damaged UHPC cubes. After the data was recorded at 28 days, both specimen conditions were evaluated upon exposure to seawater for 365 days, while only the undamaged condition was measured at 550 days. Differences in resistivity began to appear after 28 days, influenced by the types of reinforcing fibers and the hydration of the cementitious materials. Bulk resistivity ranged from 35 Ωm to 135 Ωm, with the highest values observed in Pf reinforced mixtures, specifically 10UL-25SL-Pf, 10UL-20FA-Pf, and 10UL-Pf. After 365 days, undamaged cubes exhibited significant increases in resistivity across all mixtures compared to those at 28 days, attributed to the continued hydration of PLC, FA, and SL. The percentage increases ranged from 203% for 10UL-Pf to 515% for 10UL-20FA-Sf. However, due to their conductive nature, Sf fibers limited the resistivity gains achieved through hydration and microstructural refinement [42, 43]. Consequently, the UL-FA mixture with Pf achieved roughly twice the resistivity of its steel or hybrid fiber counterparts [38]. At 550 days, further increases in bulk resistivity were observed across all mixtures, likely driven by ongoing SCM reactions. Compared to the 365-day values, resistivity increased by 15.6% to 56%. The hydration of FA and SL can reduce soluble alkalis in the pore solution and generates additional C-S–H, which fills microstructural pores and reduces connectivity. These parameters govern the electrical resistivity measurements. Consequently, mixtures containing FA and SL showed significantly improved resistivity, whereas mixtures with only UL exhibited limited improvements (although not absent), due to the limestone’s inert nature. The consistently lower resistivity of 10UL-Pf further supports this observation.

For the damaged specimens, Fig. 3 shows that after 365 days of seawater exposure, the bulk resistivity of the lab UHPC mixtures remained comparable to that of undamaged specimens of the same age, with reductions of only 3% to 25%. This suggests that seawater exposure had a limited impact on resistivity, with the reductions primarily due to cracks resulting from pre-exposure compressive strength testing. These observations suggest the possibility of similar durability between damaged and undamaged specimens. The commercial UHPC mixture showed the largest reduction in resistivity at 43%. Sf reinforced mixtures exhibited deeper cracks during the damaging process, increasing ion penetration pathways and reducing pore network tortuosity. However, due to limited information on the commercial product, its distinct behavior remains unclear.

Table 4 supports the resistivity findings through UPV results, which showed minimal differences between values at 28, 365, and 550 days. On average, UPV values changed by no more than 4.5% over time, indicating that the bulk of the specimens remained unaffected by seawater and the damage process. Even damaged samples, after 365 days, exhibited UPV values comparable to those of undamaged specimens, confirming their structural integrity. The damaged commercial UHPC showed the greatest UPV reduction, only a 6.4% decrease relative to its undamaged counterpart at the same age. Overall, the trends in UPV and resistivity align, suggesting that damaged specimens perform comparably to undamaged ones.

**Table 4:** UPV results of UHPC cubes showing pulse velocity (m/s) mean and SD

Time (days)	Condition	Comm.UHPC	10UL-20FA-Sf	10UL-20FA-SPf	10UL-20FA-Pf	10UL-25SL-Pf	10UL-Pf
28	Undamaged	5000 ± 0	4781 ± 67	4762 ± 94	4587 ± 0	4573 ± 40	4386 ± 76
365	Undamaged	4904 ± 56	4660 ± 63	4823 ± 21	4601 ± 20	4587 ± 14	4615 ± 20
	Damaged	4601 ± 20	4676 ± 76	4520 ± 53	4601 ± 35	4520 ± 59	4533 ± 55
550	Undamaged	5085 ± 24	4808 ± 16	4808 ± 24	4520 ± 62	4601 ± 20	4587 ± 8

### Compressive strength

**Fig. 4:**
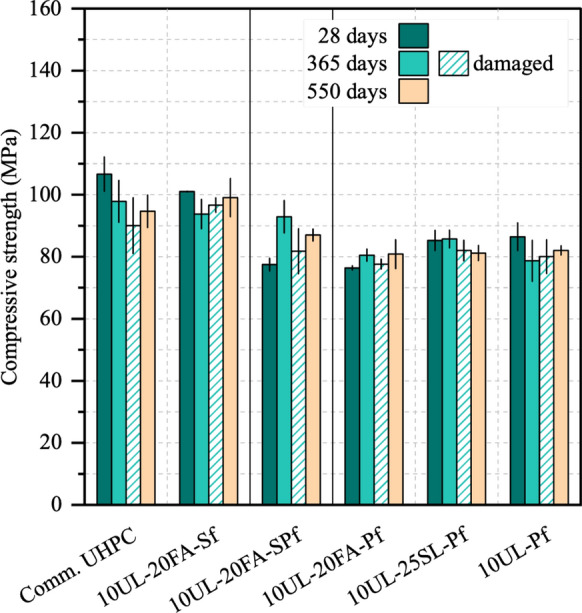
Compressive strength results of undamaged and damaged specimens at 28 days and after 365 and 550 days of exposure to seawater

The evolution of compressive strength for the various UHPC mixtures exposed to seawater is presented in Fig. 4. At 28 days, strength values ranged from 77 to 107 MPa, which are lower than typical UHPC compressive strengths. This difference can be attributed to factors such as the use of locally available materials, the high replacement of cement with SCMs, the use of PLC instead of a Type III cement, the inclusion of a relatively coarse sand, and the standard curing methods adopted. A detailed discussions of these factors is provided in [24]. Among the mixtures, the commercial UHPC achieved the highest 28-day strength, closely followed by the other Sf-reinforced mixture, 10UL-20FA-Sf. In contrast, the mixtures reinforced with Pf generally exhibited lower strength. The high aspect ratio of Pf can explain this difference. Specifically, for an equivalent fiber volume fraction, the number of Pf at the specimen’s cross-section is greater than that of Sf. This higher fiber count can disrupt the matrix packing density and increase porosity, ultimately leading to a decrease in compressive strength [44, 45]. After 365 days of seawater exposure, undamaged samples exhibited compressive strength variations from -9.3% to 18.1% relative to their 28-day values. A similar trend persisted after 550 days, with changes between − 11.8% and + 11.6% compared to the 28-day results. The mixtures 10UL-20FA-SPf, 10UL-20FA-Pf, and 10UL-25SL-Pf showed equal or higher compressive strength at 365 and 550 days compared to their 28-day reference values, with the greatest increase observed in the hybrid fiber mixture 10UL-20FA-SPf. These gains may be attributed to the continued pozzolanic reaction of fly ash and slag, which was possibly enhanced by seawater exposure [41, 46, 47]. In contrast, strength reductions were observed in the commercial UHPC, 10UL-20FA-Sf, and 10UL-Pf mixtures, which were either reinforced with Sf or contained no SCMs. For the commercial UHPC and 10UL-20FA-Sf, the reduction is likely due to superficial corrosion of steel fibers exposed to seawater. The expansion of corrosion products may have weakened the surrounding matrix and overcome the contribution of the SCMs. In the hybrid fiber mixture 10UL-20FA-SPf, this effect was not observed because of the lower Sf content (1%). Finally, for the 10UL-Pf, the strength decrease is likely related to the absence of SCMs.

**Fig. 5:**
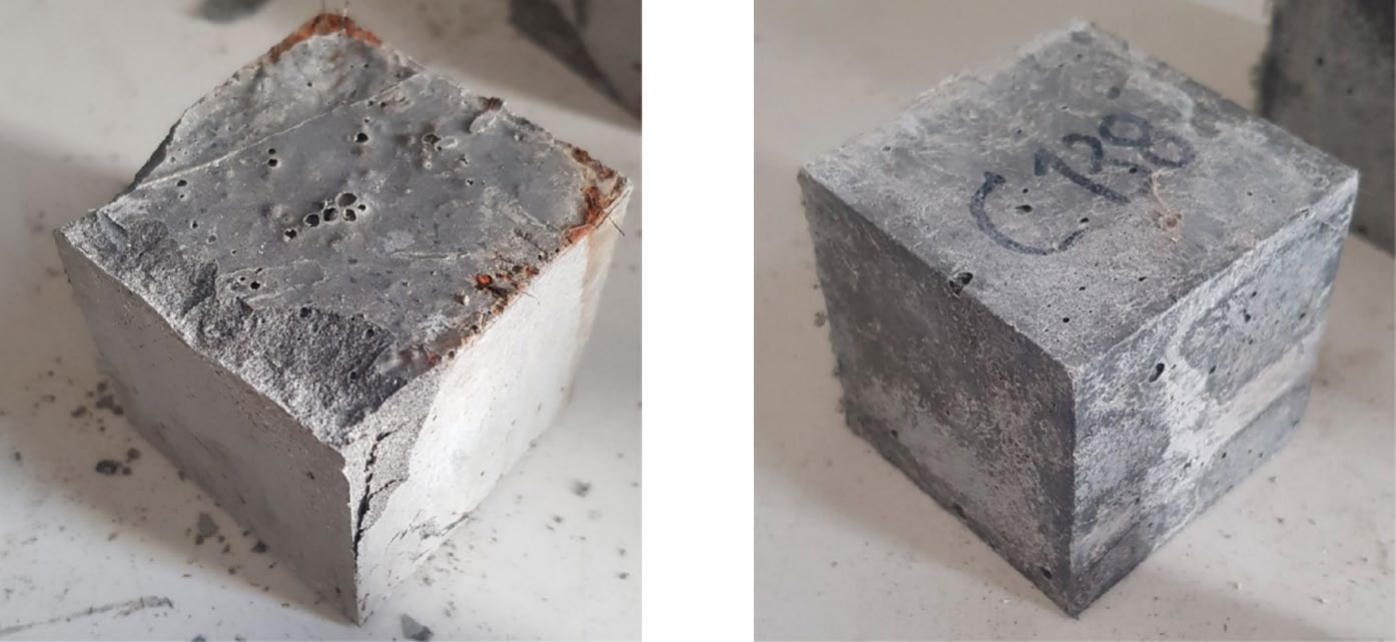
Condition of damaged UHPC cubes at 365-days seawater exposure before compressive testing. Example of a UHPC cube with Sf (left) and Pf (right)

Interestingly, despite the damaging procedure and 365 days of seawater exposure, the damaged specimens retained a high residual compressive strength, regardless of the type of reinforcing fiber used. The damaging procedure induced visible cracks and some spalling in the steel fiber-reinforced cubes, whereas the polyethylene fiber-reinforced cubes exhibited only microcracks (Fig. 5). Nevertheless, the residual strengths of these specimens ranged from − 17% to + 5% compared to their 28-day values. A possible explanation is that the extent of the damage was insufficient to develop a continuous crack pattern throughout the entire volume of the specimens. Previous studies have reported that, under uniaxial compression, the level of damage required to bridge cracks typically occurs between 70 and 90% of the concrete ultimate strength [48, 49]. When comparing damaged and undamaged specimens at 365 days, the strength values were broadly similar. Damaged mixtures reinforced with Sf exhibited an average strength reduction of 6% relative to their undamaged counterparts, whereas mixtures containing only Pf showed a smaller average reduction of 1.5%. However, these differences could in part be explained by variability, which is common in strength measurements.

Post-test visual inspection of crushed cubes confirmed the absence of corrosion on steel fibers embedded within the concrete matrix, indicating that the observed corrosion was limited only to the surface of the cubes. The fact that the embedded fibers did not corrode has implications for the UHPCs durability, suggesting that the mixtures may have developed a dense concrete microstructure that was not significantly compromised by the damaging procedure or prolonged seawater exposure.

### Silver nitrate and phenolphthalein tests

The images of the samples sprayed with the silver nitrate and phenolphthalein solutions are reported in Figure S1 (Supplementary Material), revealing no signs of chloride ingress or carbonation in any of the mixtures, regardless of the reinforcing fibers used. While the internal steel fibers showed no rusting, the rust observed on the external steel fibers was limited to areas directly exposed to seawater, as previously discussed. The lab UHPCs, and the commercial UHPC showed negligible carbonation, considering also the limited diffusion of CO_2_ in seawater. In the case of chlorides, their penetration into submerged concrete occurs primarily through diffusion, driven by the chloride concentration gradient. Unlike environments subjected to wetting–drying cycles, this process in submerged conditions stabilizes over time as the surface chloride concentration in the concrete reaches equilibrium with the surrounding seawater. The effectiveness of the lab UHPCs in resisting chloride ingress lies in their dense and discontinuous pore structure, which reduces permeability. The inclusion of Sf and Pf enhanced this effect by increasing pore tortuosity, extending the diffusion path, and slowing chloride transport. Furthermore, the content of SCMs in these mixtures further improves resistance by reducing permeability and increasing chloride-binding capacity, also because of the synergistic reactions of the SCMs with limestone.

### Water sorption

**Fig. 6:**
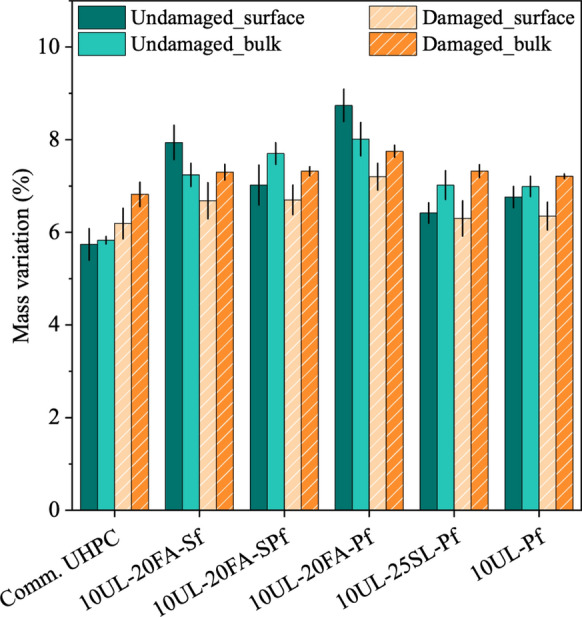
Comparison between surface and bulk mass loss measurement at 365 days of undamaged and damaged UHPC cubes

Figure 6 presents the results of the water sorption test, showing the average surface and bulk mass loss after oven drying and after 365 days of exposure for both undamaged and damaged UHPC cubes. Across all the mixtures, the mass loss values were comparable, ranging from 6.5% to 8%, only slightly higher than those of the commercial UHPC. No consistent trend was observed between surface and bulk measurements.

Surface samples, whether from undamaged or damaged specimens, did not exhibit higher average values than bulk samples, although their standard deviations were generally greater. This indicates that water ingress during exposure was negligible for all mixtures, and the observed mass variation is more likely attributable to the initial moisture content of the concrete matrix rather than to exposure conditions. These results align with the other results, suggesting that the minimal mass increases observed are linked to pore saturation at the concrete surface rather than significant ingress into the matrix. Although UHPC mixtures are expected to form a dense microstructure, it is noteworthy that even damaged specimens with visible cracks showed similar surface and bulk values. As previously discussed, in addition to the pore refinement provided by SCMs, the properties and dosage of Sf and Pf fibers acted as a physical barrier to pore connectivity and contributed to crack bridging, thereby reducing transport.

### Thermogravimetric analysis

**Fig. 7:**
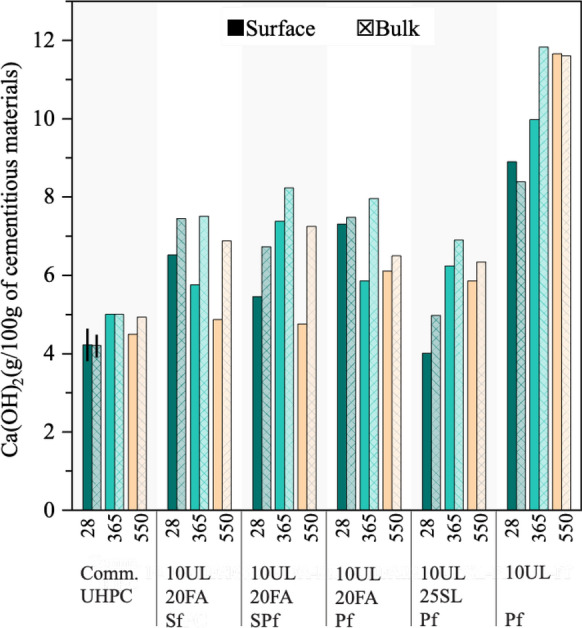
Ca(OH)_2_ results at 28 days, and after 365, and 550 days of exposure to seawater

**Fig. 8:**
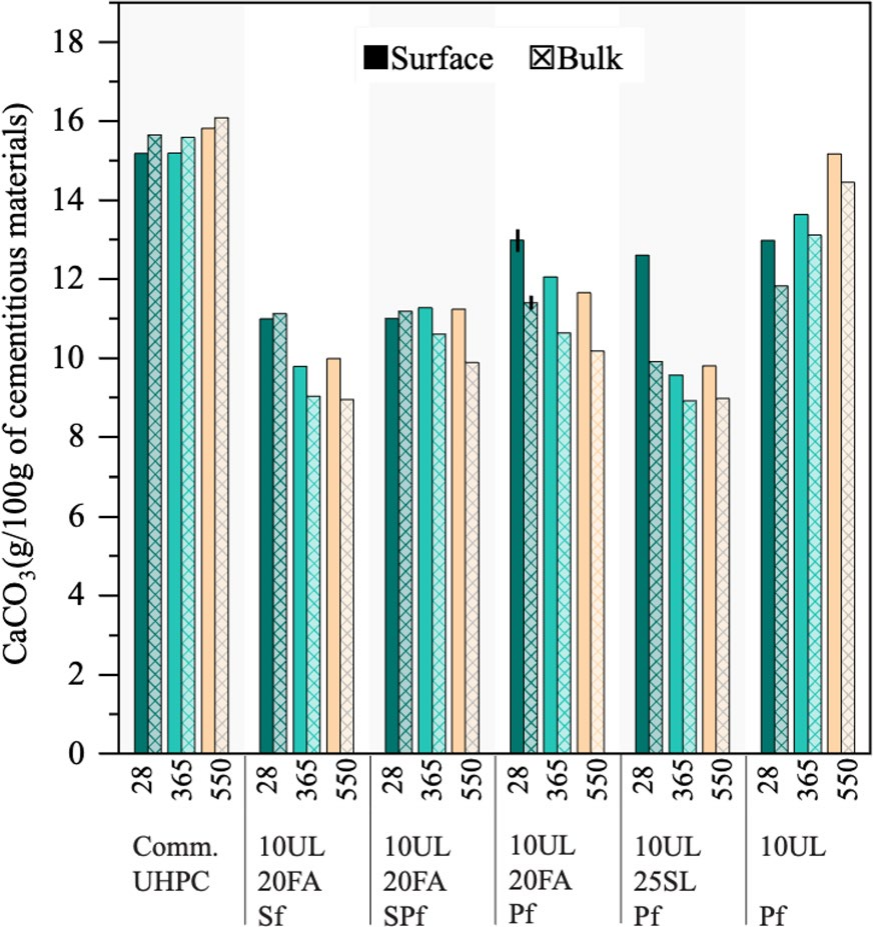
CaCO_3_ results at 28 days, and after 365, and 550 days of exposure to seawater. The error bars shown in the figure are two standard deviations of the mean

Figure 7 and Fig. 8 show the Ca(OH)_2_ and CaCO_3_ content near the surface and in the bulk of the UHPC mixtures at 28 days and after exposure to seawater at 365 and 550 days. The results of the commercial UHPC are also reported; however, the proprietary nature of its composition restricts a more comprehensive analysis of its values. The commercial UHPC showed the lowest Ca(OH)_2_ values, either because it has high volumes of SCMs, or because it has a lower cementitious content than the other mixtures. The highest Ca(OH)_2_ values at all ages were recorded by the 10UL-Pf mixture. This finding reflects what was observed in the other tests. The lack of SCMs in this formulation prevented from undergoing pozzolanic or latent hydraulic reactions, and Ca(OH)_2_ consumption did not occur. The fly ash based mixtures generally show reductions of Ca(OH)_2_ over time. On the other hand, the slag mixture showed an increase in Ca(OH)_2_ over time. Another important finding is that for all the mixtures, generally lower Ca(OH)_2_ contents were measured near the surface of the concrete samples and compared to the bulk measurements. A possible explanation for this might come from the visual observation conducted on the specimens after 365 days of seawater exposure. Some leaching was observed on the surface of the specimens, which might have contributed to the reduced Ca(OH)_2_ contents.

The CaCO_3_ TGA measurement showed similar values for all the mixtures containing FA and SL. 10UL-Pf showed the highest content among the new formulations due to the higher content of limestone in this system compared to the others. However, the lab UHPCs recorded lower values compared to the commercial UHPC. CaCO_3_ contents remained relatively constant through the different ages and exposure conditions across the mixtures confirms what was observed with the silver nitrate and phenolphthalein, i.e., the absence of carbonation in the specimens. In fact, in the FA and SL specimens, CaCO_3_ contents appear to reduce over time, possibly due to reactions between CaCO_3_ and the SCMs, although this is hard to say when running TGA on mortars.

## Conclusions

This study has demonstrated the durability and damage resistance of sustainable Portland limestone cement-based (ultra) high-performance concrete reinforced with steel fibers and polyethylene fibers when exposed to a stationary seawater environment. The key findings are summarized as follows:

- The lab UHPC mixtures exhibited minimal mass alterations during seawater exposure. After 365 days, slight leaching was observed on the surface of the specimens, but no significant mass loss/gain was recorded up to 550 days.
- The electrical resistivity of undamaged specimens significantly increased for all mixtures after seawater exposure, with supplementary cementitious materials (SCMs) contributing to these improvements, especially at later ages. Damaged samples at 365 days displayed UPV values similar to their undamaged counterparts, confirming that the bulk of the specimens remained unaffected by seawater exposure and damage processes.
- All mixtures maintained compressive strength after 365 days of exposure. Damaged samples retained residual compressive strength regardless of the fiber type. Steel fiber-reinforced cubes exhibited some visible cracks and spalling typical of high-strength concrete, while polyethylene fiber-reinforced cubes showed minimal damage, with only limited microcracks.
- No signs of chloride ingress or carbonation were observed in any of the undamaged or damaged specimens, regardless of the reinforcing fibers used. Internal steel fibers showed no rusting, while external rusting was limited to steel fibers directly exposed to seawater.
- Similar water sorption (as proxied using mass loss) values were measured in samples taken from both the surface and bulk of undamaged and damaged concrete cubes after seawater exposure.
- Lower calcium hydroxide (Ca(OH)_2_) content was measured near the concrete surface compared to the bulk, likely due to surface leaching. In addition, consistent calcium carbonate (CaCO₃) content across mixtures and exposure conditions confirmed the absence of carbonation.

These findings suggest that these sustainable UHPC mixtures exhibit enhanced resilience after more than a year and half of exposure to aggressive stationary seawater environments, even in damaged conditions. For coastal infrastructure, these UHPC mixtures demonstrate the potential to address challenges posed by climate change, slowing the aging of concrete, reducing maintenance costs, and enhancing structural resilience to damage. This increases overall safety and decreases the risk of sudden failures. Future research should investigate the performance of these mixtures under dynamic conditions in aggressive environments to further validate their applicability.

## Supplementary Information

Below is the link to the electronic supplementary material.

Supplementary file1 (DOCX 716 KB)

## Data Availability

Data will be shared on request.
