# Supplementary material for: Durability and damage resistance of sustainable Portland limestone cement-based (ultra) high-performance concrete in seawater
Source: Mater Struct. 2025 Oct 26;58(9):310. doi: 10.1617/s11527-025-02824-5 (PMC12554818; doi:10.1617/s11527-025-02824-5)
Supplement: Supplementary file 1 — Supplementary file1 (DOCX 716 KB) [file 11527_2025_2824_MOESM1_ESM.docx]

**Supplementary Material**

Table S1: Flow test results.

| Mixture | Comm. UHPC | 10UL-20FA-Sf | 10UL-20FA-SPf | 10UL-20FA-Pf | 10UL-25SL-Pf | 10UL-Pf |
| --- | --- | --- | --- | --- | --- | --- |
| Diameter_avg_ [mm] | 197±2 | 255±4 | 124±3 | 123±2 | 119±1 | 113±2 |

Table S2: Chemical composition (determined by inductively coupled plasma atomic emission spectroscopy [ICP-AES]) of the seawater, from Biscayne Bay, FL, used to immerse the specimens [1].

| Ions | Concentration, ppm |
| --- | --- |
| Calcium | 389 |
| Chloride | 18,759 |
| Iron | 0.5 |
| Potassium | 329 |
| Magnesium | 1,323 |
| Sodium | 9,585 |
| Sulfate | 2,489 |
| Nitrate | 0.1 |

a)
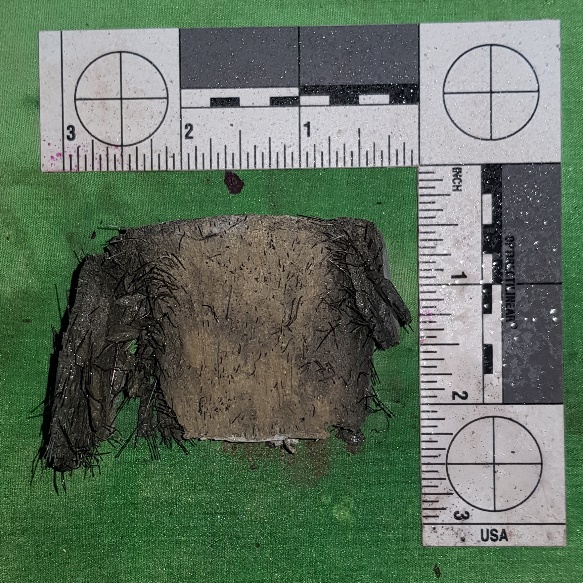
 b)
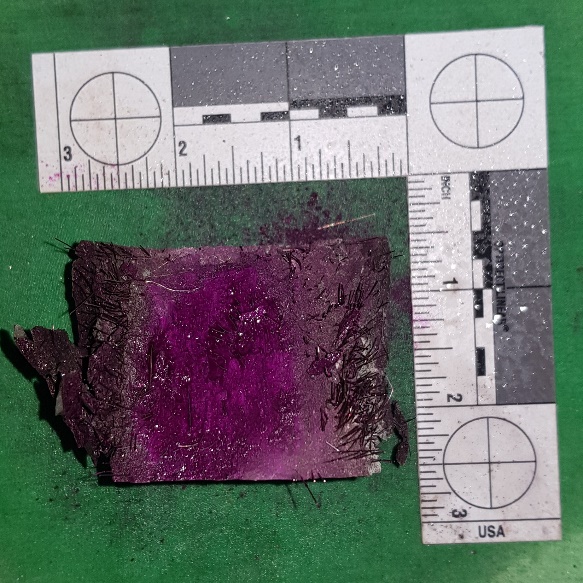


c)
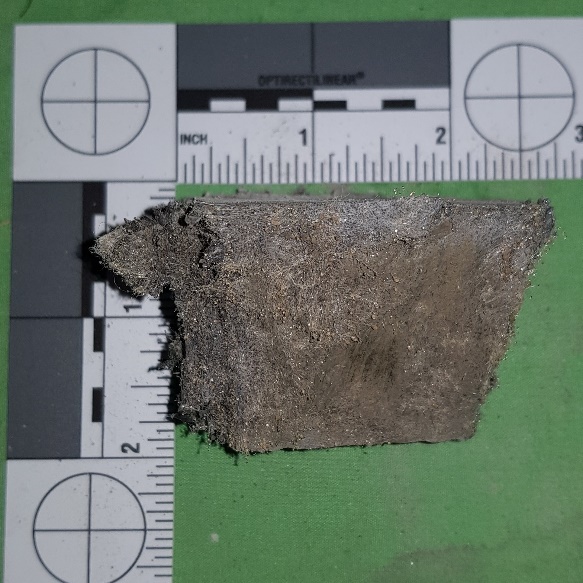
 d)
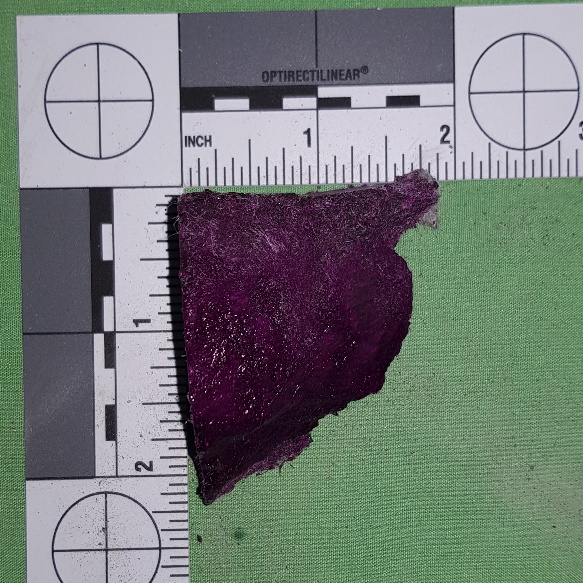


Figure S1: UHPC samples spayed with silver nitrate (a, c) and phenolphthalein (b, d). Example of Sf reinforced cubes (a, b) and Pf reinforced cubes (c, d).

**References**

1. M. Khatibmasjedi, S. Ramanathan, P. Suraneni, A. Nanni, Compressive strength development of seawater-mixed concrete subject to different curing regimes, ACI Mater J 117(5) (2020) 3-12. https://doi.org/10.14359/51725973.
